# An incidental finding of a right atrial myxoma with undiagnosed Ebstein anomaly: a case report

**DOI:** 10.1093/ehjcr/ytad539

**Published:** 2023-11-09

**Authors:** Lisa McClenaghan, Manraj Sandhu, Massimo Caputo, Paul F Brennan

**Affiliations:** Department of Adult Congenital Heart Disease, Royal Victoria Hospital, 274 Grosvenor Road, Belfast BT12 6BA, UK; Department of Cardiothoracic Surgery, Bristol Heart Institute, University of Bristol, Bristol, UK; Department of Cardiothoracic Surgery, Bristol Heart Institute, University of Bristol, Bristol, UK; Department of Adult Congenital Heart Disease, Royal Victoria Hospital, 274 Grosvenor Road, Belfast BT12 6BA, UK

**Keywords:** Case report, Myxoma, Ebstein anomaly, Tricuspid regurgitation, Echocardiography

## Abstract

**Background:**

Ebstein anomaly (EA) is a rare congenital abnormality of the tricuspid valve which can lead to progressive right heart dilatation and arrhythmias. While often seen in conjunction with other congenital cardiac lesions, such as atrial septal defects, it is not normally associated with atrial myxomas.

**Case summary:**

We present a case report of an incidental finding of a right atrial myxoma in the context of undiagnosed EA, in a 16-year-old male who presented with appendicitis. Subtle cardiomegaly on routine chest X-ray prompted further investigation, which demonstrated characteristic findings of both conditions and culminated in surgical repair using the Cone procedure. At 4-month follow-up, the patient was asymptomatic, and transthoracic echocardiography demonstrated a mean gradient of 4.5 mmHg across the tricuspid valve with mild regurgitation.

**Discussion:**

The combination of EA with right-sided myxoma is exceedingly rare, and, in this case, it may be that the apical displacement of the tricuspid valve was protective against right atrioventricular obstruction. We are reminded that although subtle abnormalities on routine investigations can be of limited significance, they can also indicate more serious underlying pathology and so consideration should be given to an appropriate cascade of further investigations to yield a timely diagnosis and enable prompt treatment.

**Learning points**

1. The combination of cardiomegaly on chest X-ray, large amplitude P waves, and apical displacement of the tricuspid valve on echocardiography should prompt clinicians to consider Ebstein anomaly.
2. In this case, the coexisting large atrial mass prompted urgent surgery. Otherwise, surgical repair of Ebstein anomaly is indicated in patients who have severe, symptomatic tricuspid regurgitation, and should be considered in patients with progressive right heart dilatation or deteriorating right ventricular systolic function.

## Introduction

Ebstein anomaly is a rare congenital abnormality of the tricuspid valve which can lead to right heart dilatation and arrhythmias. While often seen in conjunction with other congenital cardiac lesions, such as atrial septal defects (ASD), it is not normally associated with atrial myxomas.^1^ We present a case report of an incidental finding of a right atrial myxoma in the context of undiagnosed Ebstein anomaly.

## Summary figure

**Table:** 

24 February 2022	Patient presented with appendicitis and cardiomegaly noted on routine chest X-ray (CXR) on admission. Patient referred for routine outpatient cardiology review on discharge
15 June 2022	Patient reviewed in outpatient cardiology clinic. CXR repeated to ensure that rotation or poor inspiration had not affected interpretation of the original CXR; it showed persistence of cardiomegaly; therefore, an urgent outpatient echocardiogram was requested
09 September 2022	Echocardiogram revealed large pendunculated mass in the right atrium, plus Ebstein anomaly, both of which were new diagnoses. Patient transferred to tertiary cardiology centre for surgical work-up
20 September 2022	Patient transferred to specialist Ebstein surgical centre for resection of right atrial mass and repair of Ebstein anomaly
30 September 2022	Patient discharged home
9 November 2022	Repeat echocardiogram showed a stable mean tricuspid valve gradient of 4.5 mmHg, with at most mild residual tricuspid regurgitation. Patient asymptomatic on clinical review

## Case presentation

**Figure 1:**
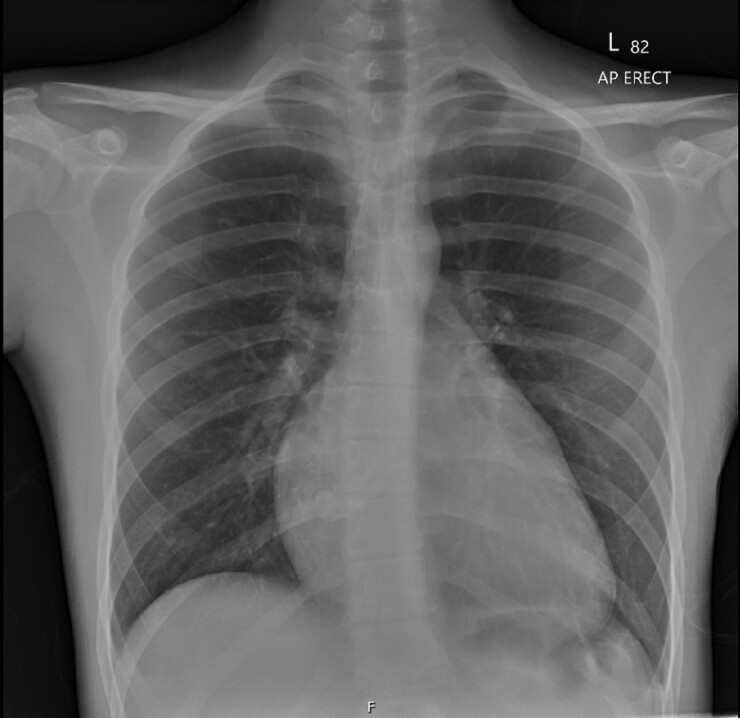
Antero-posterior chest X-ray demonstrating globular cardiomegaly.

A 16-year-old male presented with abdominal pain, and subsequently underwent a laparoscopic appendicectomy for acute appendicitis. During this admission, a routine chest X-ray (CXR) showed cardiomegaly (*Figure 1*). Following this he was referred to cardiology outpatients for routine assessment. When reviewed 4 months later, he reported 4 months of night sweats and intermittent chest pain while lying flat. His cardiovascular examination was unremarkable; he had no murmurs and there was no evidence of heart failure. He had no past medical history or family history of note and took no regular medication.

**Figure 2:**
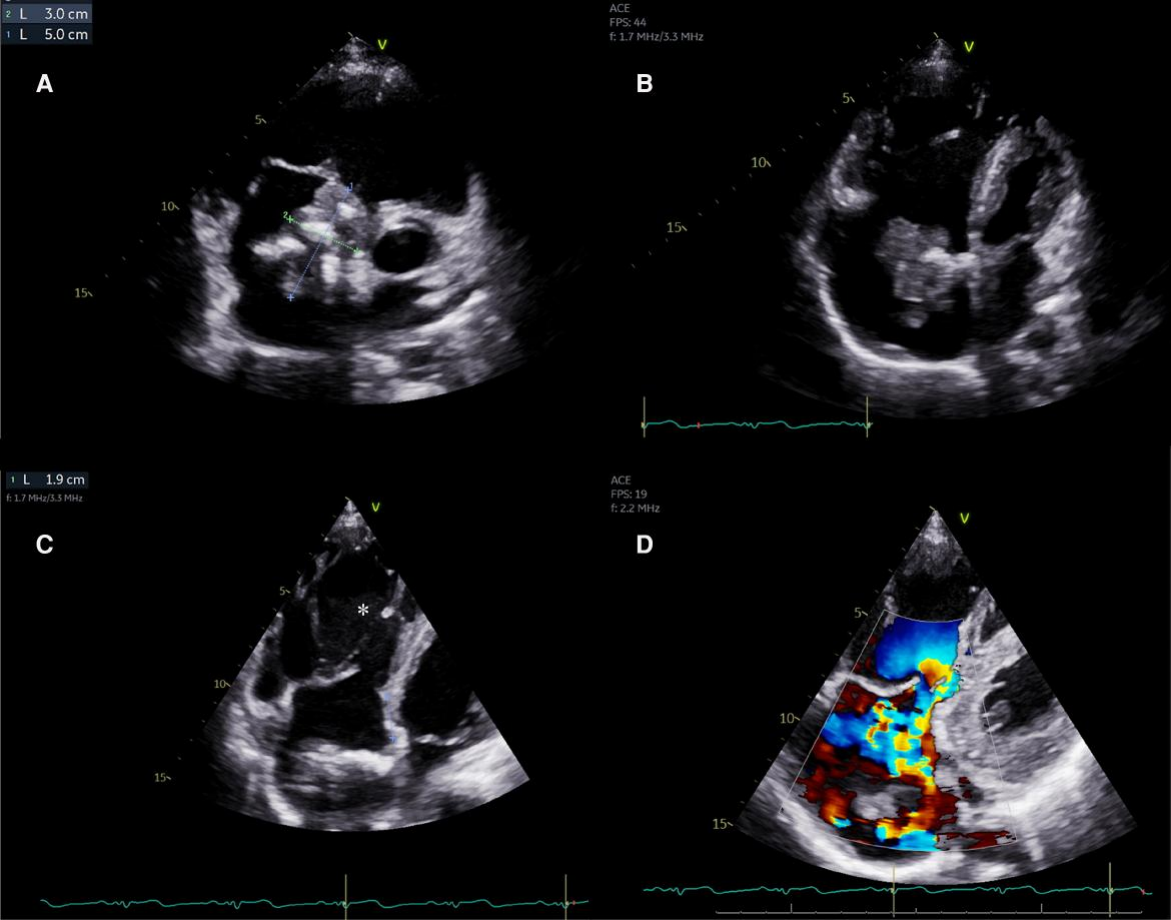
Echocardiogram showing (*A*) irregular and heterogeneous mass in an enlarged right atrium, (*B*) significant right heart dilatation, (*C*) apical displacement of the septal leaflet of the tricuspid valve (*), and (*D*) severe tricuspid regurgitation.

**Figure 3:**
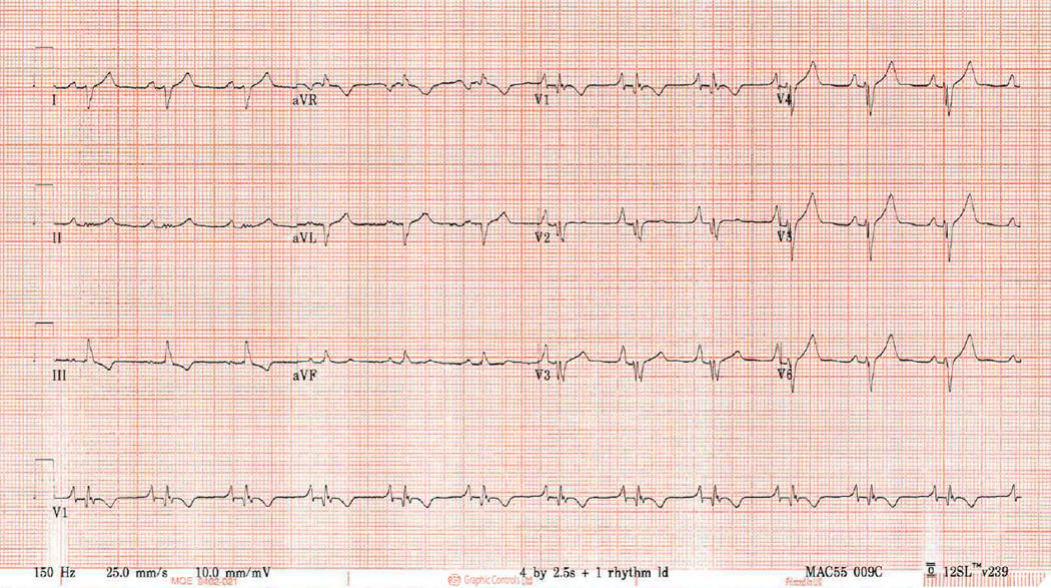
ECG showing right axis deviation and ‘Himalayan’ P waves in keeping with right atrial enlargement. ECG, electrocardiogram.

Given the persistence of cardiomegaly on repeat CXR, a transthoracic echocardiogram (TTE) was requested. This showed a large, heterogeneous, pedunculated mass in the right atrium measuring approximately 50 × 30 mm, which appeared to attach to the region of the fossa ovalis. In addition, there was apical displacement of the tricuspid valve (11.2 mm/m^2^ from mitral annulus) in keeping with undiagnosed Ebstein anomaly (EA) (*Figure 2*). The mass prolapsed through the tricuspid valve without causing obstruction (Supplementary material online, *Video S1*), and there was severe tricuspid regurgitation. The peak tricuspid regurgitant velocity was 2.2 m/s suggesting normal pulmonary artery pressure. There was no concomitant ASD. Qualitatively, the right ventricle was significantly dilated with radial impairment. Blood tests were unremarkable. His electrocardiogram (ECG) showed sinus rhythm with right axis deviation and ‘Himalayan’ P waves of large amplitude, suggestive of right atrial enlargement and typically seen in association with Ebstein anomaly (*Figure 3*). There was no evidence of pre-excitation on ECG or on telemetry. A computed tomography (CT) pulmonary angiogram showed a subacute right middle lobe pulmonary embolus, as well as more chronic, calcified emboli throughout the right pulmonary tree, for which he was commenced on low molecular weight heparin in anticipation of surgery. A CT chest, abdomen and pelvis showed no evidence of malignancy.

**Figure 4:**
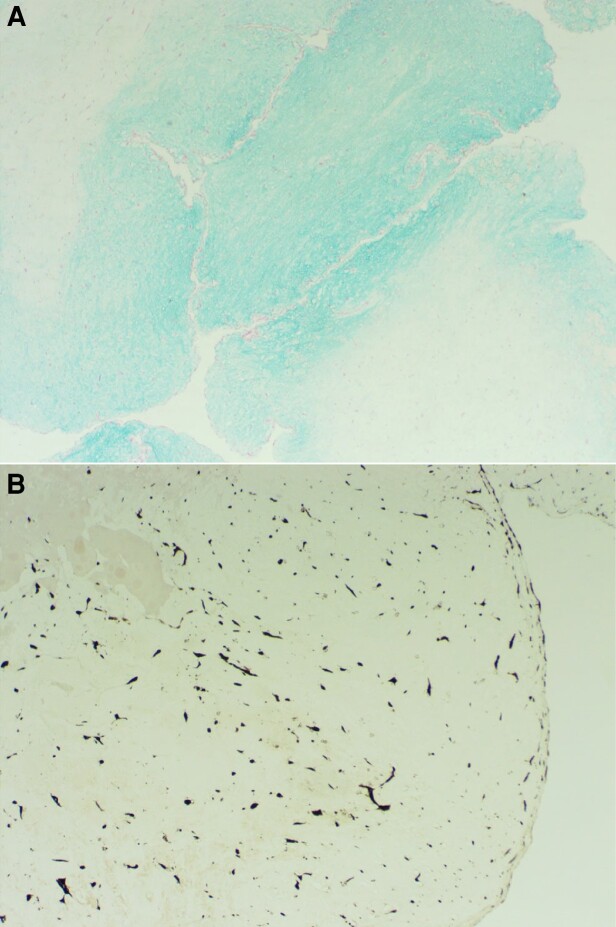
Histology slides from resected mass demonstrating (*A*) Alcian blue staining of myxoid areas and (*B*) Calretinin staining of lepidic cells.

Given the complexity of the case, our patient was referred to a specialist Ebstein surgical unit and was accepted for definitive surgery the following week. Operative appearances were suggestive of a calcified myxoma, which inserted at the foramen ovale, and this was removed along with all of the calcium on the atrial septum (Supplementary material online, *Video S2*). The tricuspid valve had a virtually absent septal leaflet, which was not delaminated, and very limited fenestrated tissue, which was not usable for the repair. The anterior and posterior leaflets were also small. The Cone repair was used;^2^ this involved detachment of the anterior and posterior leaflets from the annulus, extensive division of the sub-valvular muscle bundles and elongation of the papillary muscle, plication of the atrialized portion of the right ventricle and of the tricuspid annulus, and use of autologous pericardium to patch enlarge the anterior and posterior leaflets. Thereafter, a post-operative trans-oesophageal echocardiogram demonstrated good biventricular function with a small amount of residual tricuspid regurgitation and mild flow acceleration through the tricuspid valve (Supplementary material online, *Video S3*). Histology of the right atrial mass confirmed a 50 × 50 × 35 mm myxoma with prominent calcification (*Figure 4*). He had an uncomplicated post-operative course and, once haemostasis was achieved, anticoagulation was switched to rivaroxaban for ongoing treatment of pulmonary emboli.

His post-procedural TTE demonstrated mild tricuspid regurgitation; however, given the anatomical leaflet deficiencies, the tricuspid valve orifice was elongated and quite oblique resulting in a mean gradient, residually, of 4.8 mmHg.

At outpatient review 8 weeks later, he was asymptomatic. A repeat TTE showed a stable mean tricuspid valve gradient of 4.5 mmHg, with mild residual tricuspid regurgitation. Given that he has undergone repair of complex congenital heart disease in adulthood, he will require lifelong clinical and imaging-based evaluation to assess for long-term complications, in particular tricuspid valve dysfunction and arrhythmia.

## Discussion

Ebstein anomaly is a rare congenital abnormality of the tricuspid valve affecting approximately 1 in 200 000 live births. It is defined by five key anatomic features^1^ (*Table 1*). Commonly the tricuspid valve is regurgitant, although stenosis can also be seen. It is associated with ASD in over three quarters of cases, of which the majority shunt right to left due to elevated right heart pressures. Pulmonary stenosis and accessory pathways are also related. The absence of associated defects is highly likely to have contributed to the delayed presentation of EA in our patient.

**Table 1:** Anatomic features of Ebstein anomaly.

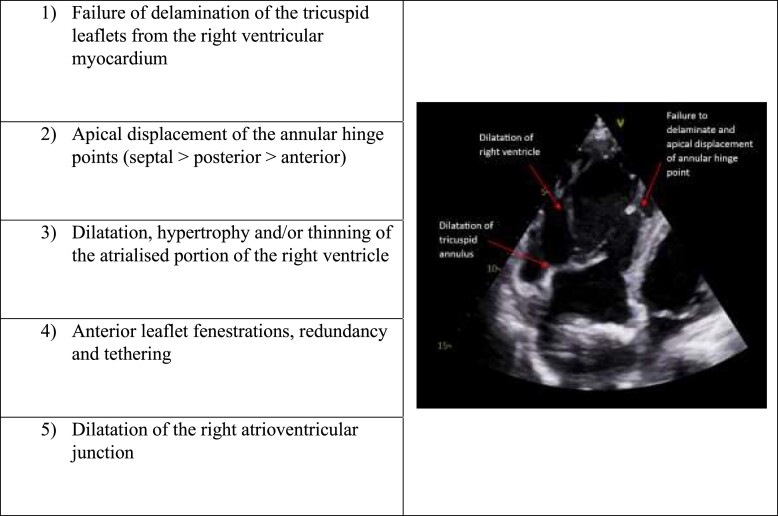

Echocardiography is the key diagnostic modality and can detect the features listed in *Table 1*. Apical displacement of the tricuspid annulus >0.8 cm/m^2^ from the mitral annulus is strongly suggestive. Often, a large ‘sail-like’ anterior leaflet can be seen, along with tethering of the septal or posterior leaflets. Magnetic resonance imaging (MRI) is a useful adjunct for volumetric and functional assessment of the right heart.

In this case, we proceeded straight to surgical repair as there was another urgent indication. Otherwise, surgery is indicated in patients with severe tricuspid regurgitation who are symptomatic, or who have objective evidence of decreased exercise capacity on exercise testing.^3^ Tricuspid valve repair is preferred over replacement and should be carried out by a surgeon with specific experience in Ebstein surgery. Progressive right heart dilatation, or evidence of deteriorating right ventricular systolic function, should also prompt consideration of surgery. In patients with a probable paradoxical embolism, ASD closure should be considered so long as they are carefully evaluated beforehand to assess the potential haemodynamic effects of doing so. There is a lifetime risk of arrhythmia; therefore, the patient should undergo regular follow-up both for imaging (echocardiography ± MRI) and ambulatory ECG monitoring.

Atrial myxomas are the most common type of primary cardiac tumour; however, as most cardiac tumours are secondary, they are relatively rare, with an incidence of 0.15/100 000/year in Ireland.^4^ Moreover, less than a quarter are found in the right atrium. Most are solitary and sporadic, although in less than 10% of cases can be familial and inherited in an autosomal dominant pattern, in which case they are more likely to be multiple. The most common sequelae are obstruction, arrhythmia, and embolic phenomena. There is wide spectrum of presentation ranging from minimal or no symptoms, as was the case with our patient, to sudden cardiac death because of arrhythmia or valve obstruction. Definitive diagnosis is histological, although certain features are highly suggestive; these include a stalk-like attachment and an attachment point at the fossa ovalis.

The combination of Ebstein anomaly with right-sided myxoma is exceedingly rare, with only two other case reports^5,6^ found in the literature, one of which was a ventricular myxoma. It may be that the apical displacement of the tricuspid valve was protective against right atrioventricular obstruction in this combination of rare cardiac pathologies. A learning point is that, although subtle abnormalities on routine investigations can be of limited significance, they can also indicate more serious underlying pathology and so consideration should be given to an appropriate cascade of further investigations. Here this helped to yield a timely diagnosis, which enabled treatment prior to the onset of frank symptoms or complications.

## Supplementary Material

ytad539_Supplementary_Data

## Data Availability

The data underlying this article are available in the article and in its Supplementary material online.

## Lead author biography

Lisa McClenaghan graduated from the University of Southampton in 2012. She is a cardiology registrar and Clinical Fellow in Adult Congenital Heart Disease at the Royal Victoria Hospital, Belfast. She is also interested in obstetric cardiology and multi-modality cardiac imaging.

## Supplementary material

**Consent:** The patient has provided written consent for publication, in accordance with the COPE guidelines.

**Funding:** None declared.
